# Activation of caspases and inhibition of ribosome biogenesis mediate antitumor activity of Chijongdan in A549 non-small lung cancer cells

**DOI:** 10.1186/1472-6882-14-420

**Published:** 2014-10-27

**Authors:** Bo Geun Kim, Hee Young Kwon, Eun Jung Sohn, Sungmin Hwang, Oh sung Kwon, Sung-Hoon Kim

**Affiliations:** College of Korean Medicine, Kyung Hee University, Hoegidong, Dongdaemungu, Seoul, 130-701 Republic of Korea

**Keywords:** Chijongdan, A549 non-small lung cancer cells, Caspase activation, Ribosomal biogenesis

## Abstract

**Background:**

Though herbal medicines have been used for cancer prevention and treatment, their scientific evidences still remain unclear so far. Thus, complementary and alternative medicine (CAM) project has been actively executed to reveal the scientific evidences in the USA and other countries. In the present study, we elucidated antitumor mechanism of Chijongdan, an oriental prescription of *Rhus verniciflua*, processed *Panax ginseng*, *Persicaria tinctoria* and *Realgar*, that has been traditionally applied for cancer treatment in Korea.

**Methods:**

Chijongdan was prepared with extracts of *Rhus verniciflua*, processed *Panax ginseng*, *Persicaria tinctoria* and processed *Realgar*. The cytotoxicity of Chijongdan was measured by MTT colorimetric assay. Cell cycle analysis was performed by FACS. Western blot was performed to see the apoptosis related proteins.

**Results:**

Chijongdan significantly exerted cytotoxicity in A549, H460 and H1299 non-small cell lung carcinoma (NSCLC) cells by MTT assay and also increased the number of ethidium homodimer positively stained cells in A549 NSCLC cells. Also, cell cycle analysis showed that Chijongdan increased sub-G1 population in a concentration dependent manner in A549 cells. In addition, Western blotting revealed that Chijongdan activated cleaved PARP, and caspase 9/3, while attenuated the expression of survival genes such as Bcl-2, Bcl-_XL_ and survivin in A549 cells. Furthermore, Chijongdan suppressed the expression of ribosomal biogenesis related proteins such as upstream binding factor (UBF), Fibrillarin, NPM (B23) and Importin-7 (IPO7) and conversely pan-caspase inhibitor Z--VAD-FMK reversed the apoptotic ability of Chijongdan to cleave PARP and caspase 3 and attenuate the expression of UBF and Fibrillarin in A549 cells.

**Conclusions:**

These findings suggest that Chijongdan induces apoptosis and inhibits ribosomal biogenesis proteins via caspase activation.

## Background

Though several therapeutic approaches such as surgery, chemotherapy, and radiotherapy have been effectively applied for cancer therapy, cancer is still one of intractable diseases all over the world [1, 2]. Apoptosis, so called programmed cell death (PCD), is characterized by specific morphological features of cell shrinkage, nuclear condensation and fragmentation and membrane blebbing in the cells [3]. It usually includes two classical apoptotic pathways such as the extrinsic death receptor pathway and intrinsic mitochondrial pathway. The extrinsic pathway is triggered by plasma membrane death receptor with its extracellular ligand Fas-L to activate caspase 8/3, while intrinsic pathway releases cytochrome c to form apoptosome to activate caspase 9/3 in the cells [4]. Thus, recently apoptosis is one of important targets in cancer therapy [4, 5] and also some herbal medicines are attractive for cancer prevention and therapy with little toxicity or combination supplement with anticancer agents [6–8].

Ribosome biogenesis is the process of making ribosomes in the cytoplasm and nucleolus to coordinate the synthesis and processing of rRNAs, and make the assembly of those rRNAs with the ribosomal proteins to maintain cell homeostasis and survival [9]. Also, there are accumulating evidences that altered ribosomal proteins [10, 11] and nuclear proteins [12–14] such as Twist, upstream binding factor (UBF), Fibrillarin, nucleophosmin (NPM, B23), exportin1 (XPO1) and importin7 (IPO7) are involved in cancer progression [15]. In contrast, some ribosomal proteins such as L13a, rp22 and rp23 were reported to function as cell cycle checkpoints and compose a new family of cell proliferation regulators [9].

Chijongdan, consisting of *Rhus verniciflua*, processed *Panax ginseng*, *Persicaria tinctoria* and *Realgar*, has been traditionally applied for cancer treatment in Korea. Nevertheless, the scientific evidence of Chijongdan is still lacking except one clinical case report [16] that Chijongdan retarded tumor size of the patient with colorectal cancer without side effect until now. Thus, in the present study, the underlying antitumor mechanism of Chijongdan using minimum dose (1%) of *Realgar* to reduce its toxicity and enhance synergistic antitumor activity with other constituent herbs was elucidated in association with apoptosis and ribosomal proteins mainly in A549 non-small lung carcinoma cells.

## Methods

### Preparation of Chijongdan

Chijongdan, consisting of three herbs and one mineral such as *Rhus verniciflua*, processed *Panax ginseng*, *Persicaria tinctoria* and processed *Realgar*, was prepared in 95% ethanol. Briefly, three herbs and one mineral were obtained from Korean Medical Hospital of Kyunghee University (Seoul, Korea) and kindly authenticated by Dr. Nam-In Baek (Department of Oriental Herbal Materials, Kyunghee University). Its four constituents (*Rhus verniciflua*, *Panax ginseng*, *Persicaria tinctoria* and processed *Realgar*), which were stored at Cancer Prevention Material Development Research Center (CPMDRC, Korea), and soaked with ethanol (2 liters × 3 changes) at room temperature for 7 days. The extract was filtered through filter paper (pore size, 3 μm), evaporated (Rotary Evaporator, model NE-1, Japan), and lyophilized (Freeze Dryer, Lioalfa-6, Telstar, Terrassa, Spain) to produce dry powder. Then, the extracts of Chijongdan were mixed together with the ratio of Chijongdan constituents (*Rhus verniciflua*, *Panax ginseng*, *Persicaria tinctoria*, *Realgar* = 60:30:9:1%).

### Cell culture

A549, H460, and H1299 cells were obtained from American Type Culture Collection (ATCC). The cells were cultured in RPMI 1640 (Welgene, Daegu, Korea) supplemented with 10% fetal bovine serum (FBS) (Welgene, Daegu, Korea) and 1% antibiotics at 37°C in a humidified 5% CO_2_ atmosphere.

### Cytotoxicity assay

The cytotoxicity of Chijongdan and its four constituents was measured in A549, H460 and H1299 lung adenocarcinoma cells by MTT colorimetric assay. The cells were seeded onto 96-well microplates at a density of 1 × 10^4^ cells per well and treated with various concentrations of Chijongdan, *Rhus verniciflua*, processed *Panax ginseng*, *Persicaria tinctoria* and *Realga*r for 24 ~ 48 h. MTT working solution (5 mg/ml in PBS) was added to each well and incubated at 37°C for 2 h. The optical density (OD) was then measured at 570 nm using a microplate reader (Sunrise, TECAN, Männedorf, Switzerland). Cell viability was calculated as a percentage of viable cells in Chijongdan, *Rhus verniciflua*, processed *Panax ginseng*, *Persicaria tinctoria* and *Realgar*-treated group versus untreated control by the following equation.


### Ethidium homodimer assay

To measure cell death, we used DAPI and ethidium homodimer dye following the manufactures’ instructions (Molecular Probes). In brief, A549 cells were treated with 100 and 200 μg/ml of Chijongdan for 24 h. After incubation, cells were fixed in 4% methanol-free formaldehyde solution, stained with the 5 μM ethidium homodimer and then incubated at 37°C for 30 min in the dark. Then the cells were mounted with mounting medium containing DAPI and then visualized under a Fluoview FV10i confocal microscope (OLYMPUS, Tokyo, Japan).

### Cell cycle analysis

A549 cells treated with Chijongdan (50, 100, 200 and 400 μg/ml) for 24 h were fixed in 75% ethanol at −20°C, resuspended in PBS containing RNase A (1 mg/ml), and incubated for 1 h at 37°C. The fixed cells were stained with propidium iodide (50 μg/ml) for 30 min at room temperature in dark. The DNA contents of the stained cells were analyzed using CellQuest Software with the FACSCalibur flow cytometry (Becton Dickinson, Franklin Lakes, NJ).

### Western blotting

Whole cell lysates from the cells exposed to Chijongdan (25, 50, 100 and 200 μg/ml) for 24 h were prepared using RIPA buffer (50 mM Tris–HCl, pH 7.5, 150 mM sodium chloride, 1% Triton X-100, 0.1% SDS, 2 mM EDTA, 0.5% sodium deoxycholate). The protein contents in the supernatants were measured by using a Bio-Rad DC protein assay kit II (Bio-Rad, Hercules, CA), separated on 4–12% NuPAGE Bis-Tris gels (Invitrogen, Carlsbad, CA) and electro-transferred onto a Hybond ECL transfer membrane (GE Health Are Bio-Science, Piscataway, NJ). The membranes were blocked with 5% nonfat dry milk and immunoblotted with caspase-3, caspase-9, PARP, Bcl-2, Bcl-x_L_, survivin, UBF, Fibrillarin, NPM (B23), IPO7 or β-actin (Cell signaling, Danvers, MA) antibodies.

### Statistical analyses

Statistical analysis of the data was conducted using Sigmaplot version 12 software (Systat Software Inc., San Jose, CA). All data were expressed as means ± standard deviation (SD). One-way ANOVA was used for comparison of multiple groups. Student *t*-test was used for comparison of two groups. Statistical difference was set at *p* values of <0.05 between control and Chijongdan-treated groups.

## Results

### Chijongdan and its constituents exerted the cytotoxicity against human non-small cell lung cancer cells

**Figure 1:**
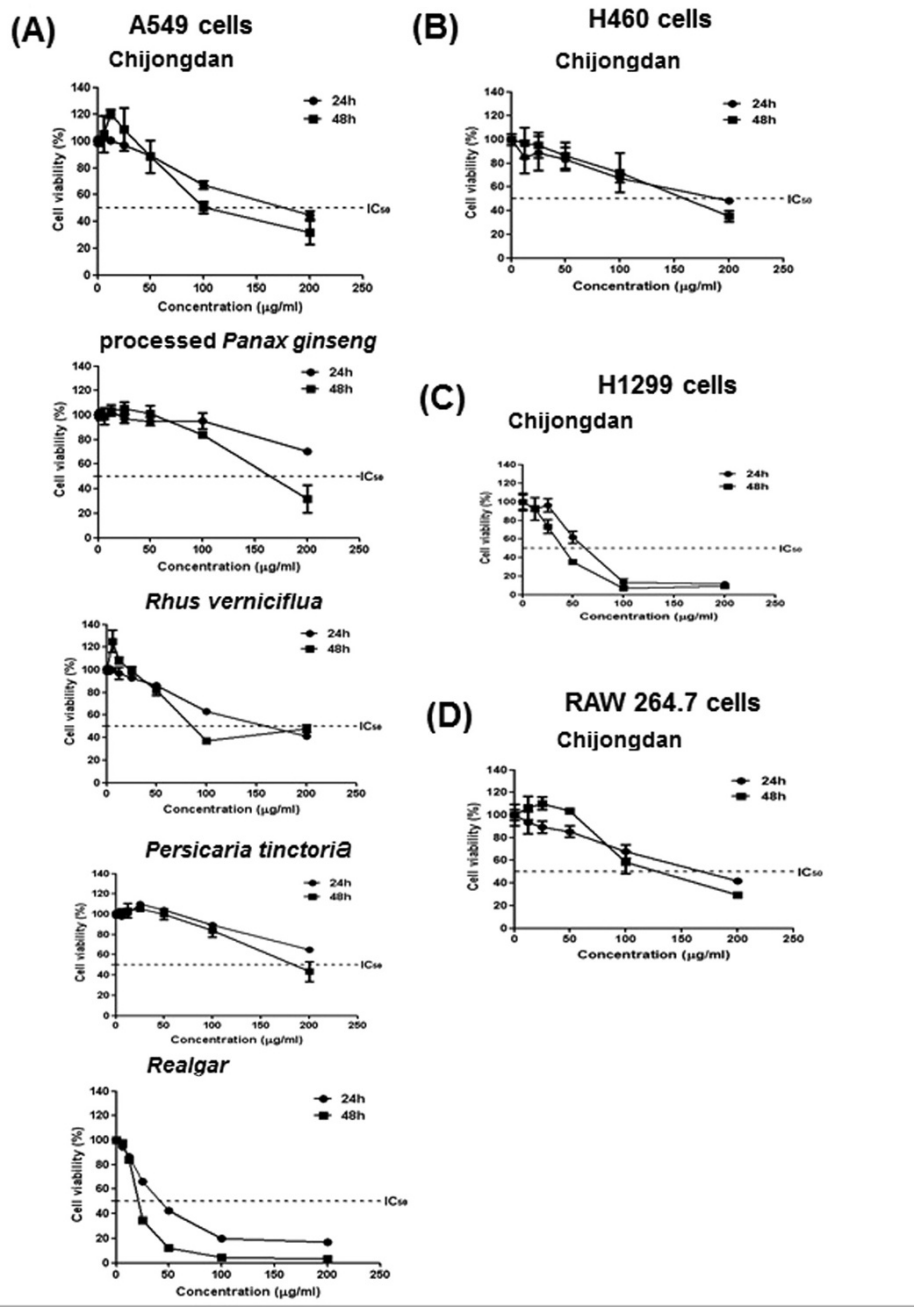
**Cytotoxicity of Chijongdan and its constituents in non**-**small cell lung carcinoma cells.** A549, H460 and H1299 cells were exposed to Chijongdan for 24 or 48 h. Then MTT assay was performed as shown in Methods. Effect of Chijongdan and the constituents on cytotoxicity in A549 **(A)**, H460 **(B)**, H1299 **(C)** and RAW264.7 cells **(D)**. All data were expressed as means ± standard deviation (SD).

To evaluate the cytotoxic effect of Chijongdan and its constituents in human non-small cell lung carcinoma cells (NSCLC), we conducted MTT assay. A549, H460 and H1299 cells were treated with various concentrations (0, 6.25, 12.5, 25, 50, 100 or 200 μg/ml) of Chijongdan and its constituents such as *Rhus verniciflua*, processed *Panax ginseng*, *Persicaria tinctoria* and processed *Realgar* for 24 h and 48 h. Chijongdan significantly decreased the viability of A549, H460 and H1299 cells (Figure 1A, B, C). However, the cytotoxicity of Chijongdan was weaker in RAW264.7 macrophage cells compared to H1299 cells (Figure 1D), but not significantly for other NSCLCs, implying somewhat cytotoxicity in normal cells. *Realgar* was more cytotoxic in A549 cells than other constituent herbs such as *Rhus verniciflua*, processed *Panax ginseng* and *Persicaria tinctoria* (Figure 1A).

### Chijongdan induced apoptotic morphology in A549 cells

**Figure 2:**
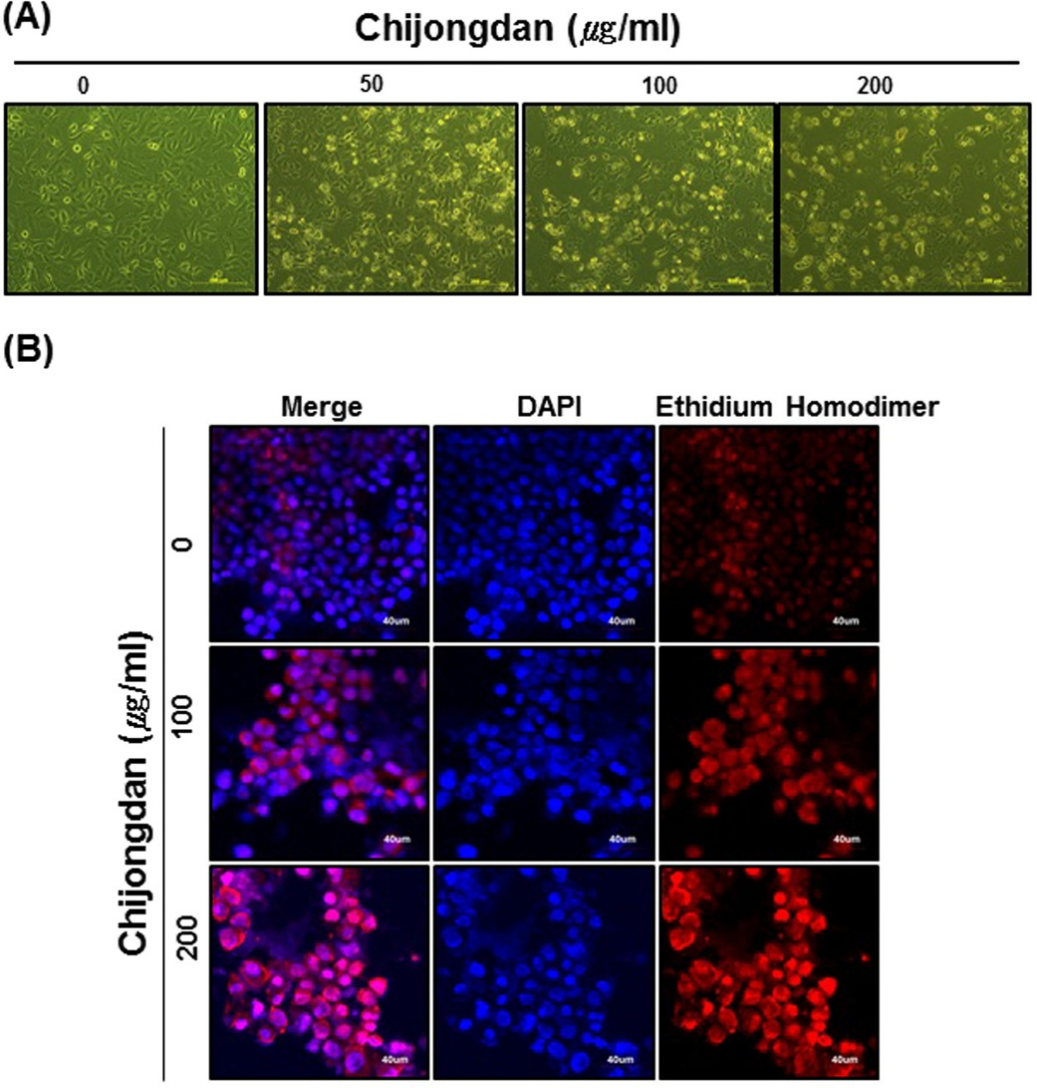
**Effect of Chijongdan on ethidium homodimer stained cells in A549 cells.** A549 cells were treated with various concentrations of 50, 100 and 200 μg/ml of Chijongdan for 24 h. **(A)** Morphology of A549 cells was observed in inverted microscope. **(B)** A549 cells were stained with DAPI and ethidium homodimer dyes, mounted with mounting medium containing DAPI and then visualized under a Fluoview FV10i confocal microscope (OLYMPUS, Tokyo, Japan).

Morphological changes in Chijongdan treated A549 cells were observed under inverted microscopy. As shown in Figure 2 A, the cells treated with 0, 50, 100 or 200 μg/ml of Chijongdan for 24 h exhibited apoptotic features such as apoptotic bodies and cell shrinkage.

### Chijongdan increased ethidium homodimer positively stained cells in A549 cells

Ethidium homodimer dye staining was performed to detect cell death. We observed apoptotic bodies in Chijongdan treated A549 cells in a dose dependent manner, indicating apoptotic feature, while intact morphology was shown in untreated control (Figure 2B).

### Chijongdan significantly increased the sub-G1 portion in A549 cells

**Figure 3:**
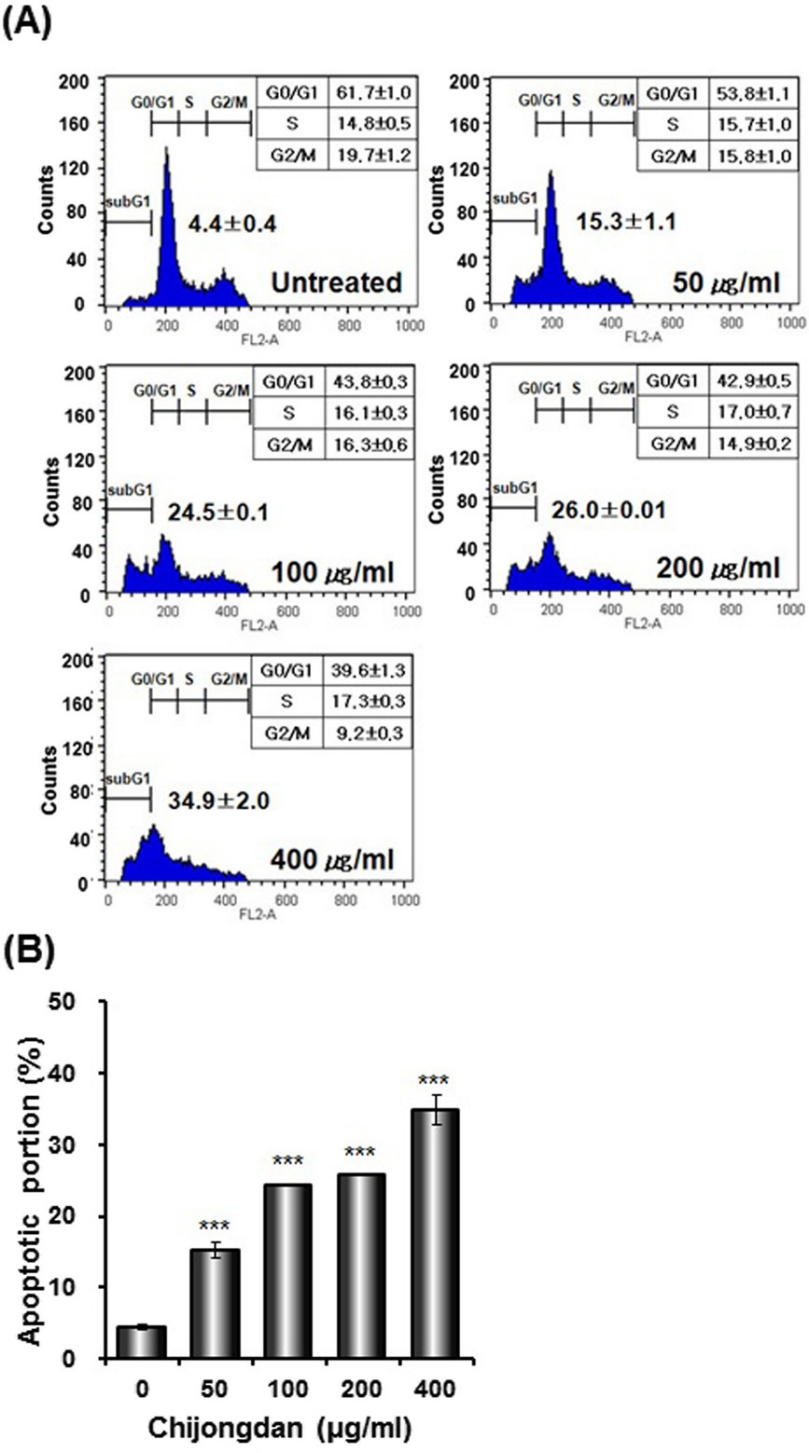
**Effect of Chijongdan on sub G1 population in A549 cells.** A549 cells were treated with 50, 100, 200 and 400 μg/ml of Chijongdan for 24 h and stained with propidium iodide (50 μg/ml) for 30 min at room temperature in dark. **(A)** The DNA contents of the stained cells were analyzed by the FACSCalibur flow cytometry. **(B)** Bar graphs represent percentages of apoptotic portion. All data were expressed as means ± standard deviation (SD). ***p < 0.001 vs. untreated control.

To investigate the effect of Chijongdan on cell cycle phase distribution, A549 cells were treated with various concentrations (0, 50, 100, 200 or 400 μg/ml) of Chijongdan. As shown in Figure 3A and 3B, Chijongdan significantly increased sub-G1 phase proportion in a dose dependent manner.

### Chijongdan regulated apoptosis related proteins in A549 cells

**Figure 4:**
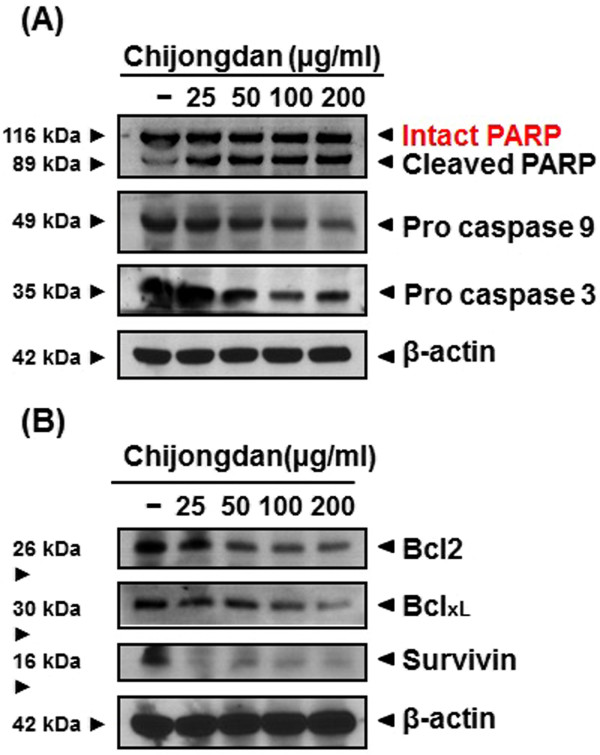
**Effect of Chijongdan on pro**-**apoptotic and anti** **-apoptotic proteins in A549 cells.** A549 cells were treated with 25, 50, 100 and 200 μg/ml of Chijongdan for 24 h and Western blotting was performed for pro-apoptotic **(A)** and anti-apoptotic proteins **(B)** as shown in Methods.

To confirm whether the cytotoxic effect of Chijongdan in A549 is associated with apoptosis, we performed Western blotting. A549 cells were treated with 25, 50, 100 and 200 μg/ml of Chijongdan for 24 h. The cell lysates were prepared and subjected to Western blotting for PARP, procasepase-3, −9, Bcl-x_L_, Bcl-2, and survivin. Consistent with previous data, anti-apoptotic proteins such as Bcl-x_L_, Bcl-2, and survivin were downregulated by Chijongdan in a dose dependent manner, while pro-apoptotic protein such as caspase-3, −9 and PARP were cleaved in A549 cells (Figure 4A,B).

### Effect of Chijongdan on apoptotic and ribosome biogenesis related proteins was reversed by pancaspase inhibitor Z-VAD-FMK in A549 cells

**Figure 5:**
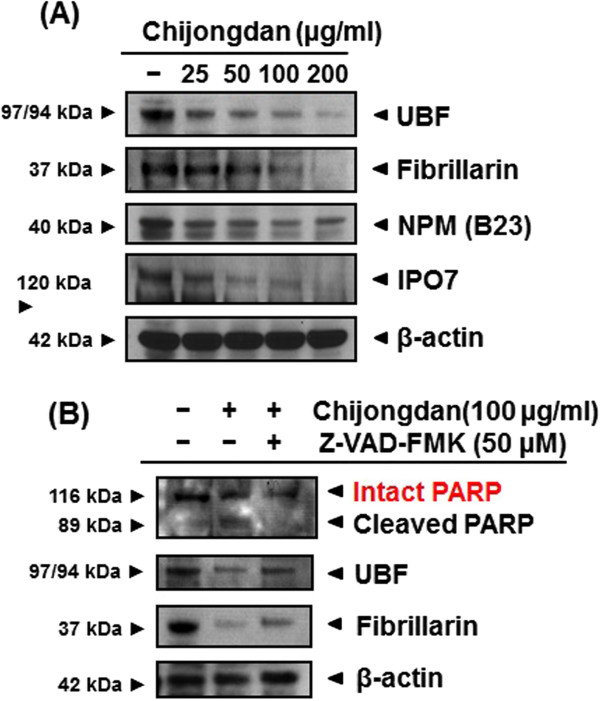
**Effect of Chijongdan on apoptotic and ribosome biogenesis related proteins was reversed by pancaspase inhibitor Z-VAD-FMK in A549 cells. (A)** Effect of Chijongdan on ribosomal biogenesis related proteins in A549 cells. **(B)** Effect of pan-caspase inhibitor Z--VAD-FMK on the ability of Chijongdan to inhibit ribosomal biogenesis and induce apoptosis in A549 cells. A549 cells were treated with 100 μg/ml of Chijongdan and/or pan-caspase inhibitor Z--VAD-FMK for 24 h and Western blotting was performed for UBF, Fibrillarin and PARP as shown in Methods.

It was well demonstrated that ribosome production was enhanced in cancer cells and that ribosome biogenesis played a crucial role in tumor progression [17]. To confirm that Chijongdan can regulate the ribosome biogenesis related proteins, we conducted Western blotting. As shown in Figure 5A, Chijongdan down-regulated UBF, Fibrillarin, NPM and IPO7 in a dose dependent fashion. Conversely, the effects of Chijongdan on ribosome biogenesis and apoptosis were reversed by pan-caspase inhibitor Z--VAD-FMK (Figure 5B), indicating that Chijongdan induces apoptosis and inhibits ribosomal biogenesis proteins via caspase activation.

## Discussion

Lung cancer is the most common cause of cancer death worldwide [18]. Among lung cancers, 80% are classified as non-small cell lung cancer (NSCLC) and 20% are small cell lung cancer (SCLC). Though recently advanced computed tomography (CT) scanning made possible detection of NSCLCs at earlier stages [19], over 70% of lung cancers are still loco-regionally advanced or metastatic at the time of diagnosis [20]. Cell proliferation needs to duplicate their own structural and functional components [21] via increased protein synthesis which, in turn, followed by an up-regulation of the ribosome biogenesis rate [21–23].

Herbal mixture Chijongdan has been traditionally used for cancer therapy without clear scientific evidences. Among constituents of Chijongdan, *Rhus verniciflua* was known to have antitumor activity in A549 lung cancer [24], MCF-7 breast cancer [25], lymphoma [26], osteosarcoma [27] and renal cancer [28] and also processed *Panax ginseng* was reported to exert antitumor effect in AGS gastric cancer. Also, *Persicaria tinctoria* contained tyrosine kinase inhibitory compounds [29] and *Realgar* was reported to be too cytotoxic and apoptotic in chronic lymphocytic leukemia or cervical cell lines [30, 31]. Here, based on previous antitumor evidences of its constituents, antitumor activity of Chijongdan was hypothesized. Thus, we investigated antitumor mechanism of Chijongdan in association with apoptosis and ribosomal proteins in non-small lung carcinoma cells. Here we found that Chijongdan and its constituents significantly exerted cytotoxicity in A549, H460 and H1299 NSCLC cells, showed morphologic changes such as apoptotic bodies cell shrinkage in a dose dependent manner and increased the number of ethidium homodimer positively stained cells in A549 cells, indicating the cytotoxicity of Chijongdan was due to apoptosis induction in A549 cells. Consistently, cell cycle analysis showed that Chijongdan increased sub-G1 population and also cleaved PARP, activated caspase9/3, and attenuated the expressed of survival genes such as Bcl-2, Bcl-x_L_ and survivin in A549 cells, implying Chijongdan induces apoptosis and suppresses survival genes. Furthermore, Chijongdan repressed the expression of ribosome biogenesis related proteins such as UBF, Fibrillarin, NPM, and IPO7, indicating that Chijongdan can inhibit the rDNA transcription or processing of rRNA during cancer progression of A549 cells. Similarly, melatonin suppressed 45S preribosomal RNA and UBF and enhanced the antitumor activity of puromycin in MDA-MB 231 breast cancer cells [32]. Interestingly, the antitumor ability of Chijongdan to cleave PARP and caspase 3 and inhibit the expression of UBF and Fibrillarin was blocked by pan-caspase inhibitor Z-VAD-FMK in A549 cells, demonstrating that caspase activation mediates the inhibition of ribosomal biogenesis by Chijongdan in A549 cells.

## Conclusions

Taken together, these findings suggest that Chijongdan induces apoptosis and inhibits ribosomal biogenesis proteins via caspase activation in A549 non-small cell lung carcinoma cells, though it still needs further research including *in vivo* efficacy and safety and pharmacodynamic studies with Chijongdan and its constituents in the future.
